# Impact of cultivar on grapevine physiological adaptation to drought and subsequent rehydration

**DOI:** 10.3389/fpls.2026.1757730

**Published:** 2026-03-27

**Authors:** Sara Pereira, José Moutinho-Pereira, Carolina Maia, Renata Moura, Cátia Brito, Lia-Tânia Dinis, Sandra Pereira

**Affiliations:** 1 Centre for the Research and Technology of Agro-Environmental and Biological Sciences (CITAB), University of Trás-os-Montes e Alto Douro (UTAD), Vila Real, Portugal; 2 Institute for Innovation, Capacity Building and Sustainability of Agri-Food Production (Inov4Agro), University of Trás-os-Montes e Alto Douro (UTAD), Vila Real, Portugal

**Keywords:** climate change adaptation, Douro region, Tinto Cão, Touriga Franca, *Vitis vinifera*, water deficit

## Abstract

**Introduction:**

Understanding grapevine responses to water deficit and rewatering is essential for developing adaptive strategies to mitigate the negative impacts of climate change in Mediterranean viticulture.

**Methods:**

This study evaluated the ecophysiological and biochemical behaviour of two emblematic Douro cultivars, Touriga Franca (TF) and Tinto Cão (TC), subjected to contrasting water regimes under potted controlled conditions. Physiological measurements of gas exchange, chlorophyll *a* fluorescence, relative water content (RWC), and leaf biochemical parameters (photosynthetic pigments, phenolic compounds, flavonoids, soluble proteins, soluble sugars, and antioxidant activity) were performed after drought and recovery phases. Results revealed distinct adaptive responses between cultivars.

**Results and discussion:**

TF exhibited moderate stomatal regulation, maintaining photosynthetic activity and high intrinsic water use efficiency during drought, as well as rapid recovery after rehydration. Conversely, TC showed a more conservative strategy, characterized by earlier stomatal closure, higher water retention (RWC > 90%), and increased accumulation of soluble proteins and phenolic compounds under stress. Both cultivars activated photoprotective mechanisms, with TF showing enhanced non-photochemical quenching and TC maintaining stable photosystem II efficiency. These findings highlight the contrasting strategies of TF (more anisohydric, plastic, and rapidly recoverable) and TC (more isohydric, conservative, and resilient), offering valuable insights for the selection of drought-tolerant cultivars and sustainable management of Douro vineyards under future warming and aridity scenarios.

## Introduction

The grapevine (*Vitis vinifera* L.) plays a major economic and cultural role, particularly in Mediterranean regions. Worldwide, viticulture represents a highly valuable agricultural sector, occupying millions of hectares and contributing significantly to international trade. According to the International Organisation of Vine and Wine (OIV, 2024), global wine production reached a historically low level of approximately 237 million hectoliters in 2023, with projections indicating a further decline to 225.8 million hectoliters in 2024 (OIV, 2025).

The Douro Demarcated Region (DDR), established in 1756 as the world’s first officially regulated wine region, stands out for its extensive territory of around 250,000 hectares along the Douro Valley and its tributaries, and for its marked edaphoclimatic diversity. The region is divided into three sub-regions (Baixo Corgo, Cima Corgo and Douro Superior), whose heterogeneous soils and Mediterranean climate, characterised by hot, dry summers and cold, wet winters, strongly influence grapevine growth, development and wine quality (Jones and Alves, 2012). High summer evapotranspiration, combined with irregular rainfall, leads to pronounced water deficits during the growing season, thereby reducing both yield and grape quality (Malheiro et al., 2007). This constraint is expected to intensify in the coming decades, as climate projections indicate reductions in soil water availability of up to 70% across the Iberian Peninsula (Stigliani and Salomons, 1992; Schultz, 2000). Since the wine quality begins in the vineyard, depending on grapevine cultivars, rootstocks, and environmental conditions (Aubert and Chalot, 2018; Gambetta et al., 2020), climate change has become a critical factor. Climate variability and warming influence grapevine physiology, affecting yield and berry composition (Dinis et al., 2018; Rogiers et al., 2022). Environmental stresses also modulate the accumulation of phenolic compounds, anthocyanins and other antioxidants, which are essential to wine sensory properties, color, flavor and oxidative stability (Mu et al., 2016; Fabani et al., 2017; Gutiérrez-Escobar et al., 2021; Vo et al., 2022) and play a protective role against reactive oxygen and nitrogen species (Mao et al., 2017). Generally, moderate water deficits enhance phenolic content and aromatic complexity, whereas severe deficits compromise yield, ripening and wine composition (Ojeda et al., 2002; Deloire et al., 2004; Van Leeuwen et al., 2009). Rising temperatures and irregular rainfall further accelerate phenological development, resulting in excessive sugar accumulation and the degradation of organic acids (Coombe, 1987; Bock et al., 2011; Fraga et al., 2012; Alikadic et al., 2019). Reduced stomatal conductance limits CO_2_ assimilation and photosynthesis, and under extreme stress, Rubisco activity may also decline, favoring photorespiration (Griffin and Seemann, 1996; Long et al., 2006; Dinis et al., 2014). It is also well established that responses to water stress are cultivar-specific. Isohydric cultivars maintain relatively stable water potentials to prevent dehydration, whereas anisohydric cultivars sustain higher rates of CO_2_ assimilation at the expense of increased water loss (Poni et al., 2007; Chaves et al., 2016). Hormonal signaling, particularly the action of abscisic acid (ABA), plays a central role in these responses, regulating stomatal closure, water balance and berry ripening (Fortes et al., 2015; Gomez-Cadenas et al., 2015; Dinis et al., 2018).

In this context, the need to adapt viticulture to climate change has stimulated research on adaptation strategies and on the intraspecific diversity of grapevine (Duchêne et al., 2010; Myles, 2013; Carvalho et al., 2017; Morales-Castilla et al., 2020). Understanding varietal responses to environmental stress helps identify cultivars that are more resilient to future climate scenarios and traits of interest for breeding programmes (Morales-Castilla et al., 2020). These adaptation strategies encompass coordinated actions, methods and practices aimed at mitigating the negative impacts of climate change on grapevine performance and wine quality (Touzard and Ollat, 2014).

This study focuses on two red cultivars, Touriga Franca and Tinto Cão, both grafted onto the 1103 Paulsen (1103 P) rootstock, widely recognized for its tolerance to hot and dry conditions (Magalhães, 2008). Touriga Franca is one of the most relevant and widely planted cultivars in the Douro, known for its consistent productivity and physiological robustness (Magalhães, 2008; Böhm, 2010). Tinto Cão, although historically less widespread, has been increasing in regional importance due to the high quality of the wines it produces and its marked tolerance to heat and drought stress (Magalhães, 2008; Böhm, 2010; Moutinho-Pereira et al., 2015; Santos et al., 2019). Studies further indicate that this cultivar remains well adapted under future warming scenarios, partly due to its distinctive chlorophyll pigment profile, which may enhance resilience to high radiation and combined heat–drought conditions (Moutinho-Pereira et al., 2015).

Thus, this work aims to evaluate the ecophysiological behavior of Touriga Franca and Tinto Cão, grafted onto 1103 P, under different water regimes, contributing to the development of adaptation strategies for Douro viticulture in a context of increasing aridity.

## Materials and methods

### Plant material and experimental design

The experimental trial was conducted from April to August 2024 at the facilities of the University of Trás-os-Montes and Alto Douro (UTAD), in Vila Real (41°17’18”N; 7°44’12”W), located in the Baixo Corgo sub-region of the Douro Demarcated Region (RDD), northern Portugal. Meteorological data recorded between April and September 2024 were obtained from a weather station located on the UTAD campus.

Two autochthonous *Vitis vinifera* L. cultivars, Tinto Cão (TC) and Touriga Franca (TF), grafted onto the 1103 Paulsen (1103 P) rootstock, were used. The plants consisted of grafted nursery vines of uniform age and physiological development at the time of the experiment. All plants were obtained from the same propagation batch to ensure homogeneity of plant material. The experiment was established in pots, with a volume of 15 L, and subjected to a homogeneous fertilization regime to prevent potential interferences arising from nutrient deficiencies in physiological responses. The substrate consisted of a 2:1:1 mixture of vineyard soil, peat and perlite. The vineyard soil component was classified as an Eutric Cambisol (IUSS Working Group WRB, 2022) and was collected from the 0–20 cm horizon at the University of Trás-os-Montes e Alto Douro (UTAD). Following collection, the soil was air-dried and sieved to 5 mm in preparation for substrate formulation.

All physicochemical characterization of the soil was performed at the Soil Laboratory of UTAD, following standard analytical procedures. The soil presented a coarse texture and a moderately acidic reaction, with pH H_2_O ranging from 6.3 to 6.4 and pH KCl from 4.7 to 4.8. It had a low organic matter content (0.5–0.8%), medium levels of extractable phosphorus (51–76 mg P_2_O_5_ kg^−1^) and high extractable potassium concentrations (116–149 mg K_2_O kg^−1^). The effective cation exchange capacity was low (CTC_e_ ≈ 4.7–5.8 cmolc kg^−1^), with calcium representing the predominant exchangeable cation, although at comparatively low absolute concentrations.

Each plant received a total of 1.25 g of NPK (12-11-18) fertilizer, supplemented with 0.25 g of potassium chloride (KCl, 60% K_2_O) and 3.0 g of triple superphosphate (45% P_2_O_5_). Fertilization was applied once, at the beginning of the trial on April 15, and incorporated into the upper layer of the substrate to ensure a balanced and continuous supply of nutrients throughout plant development.

Each pot containing one grapevine plant was considered an independent biological replicate. A total of 12 plants per cultivar were used, corresponding to six biological replicates per irrigation regime. Physiological measurements and biochemical analyses were performed using these biological replicates. Initially, all plants were irrigated to field capacity to allow a uniform acclimation period. On July 3, water deficit induction began in half of the plants of each cultivar (six pots per cultivar), while the remaining plants continued to be irrigated regularly, constituting the control group. The drought period lasted from July 3 to July 15, during which the water-stressed plants received no irrigation until maintaining the stomatal conductance at 50 mmol·m^−2^·s^−1^, whereas the control plants were irrigated three times per week to maintain well-watered conditions close to substrate field capacity, ensuring the absence of water limitation throughout the experimental period.

**Figure 1:**
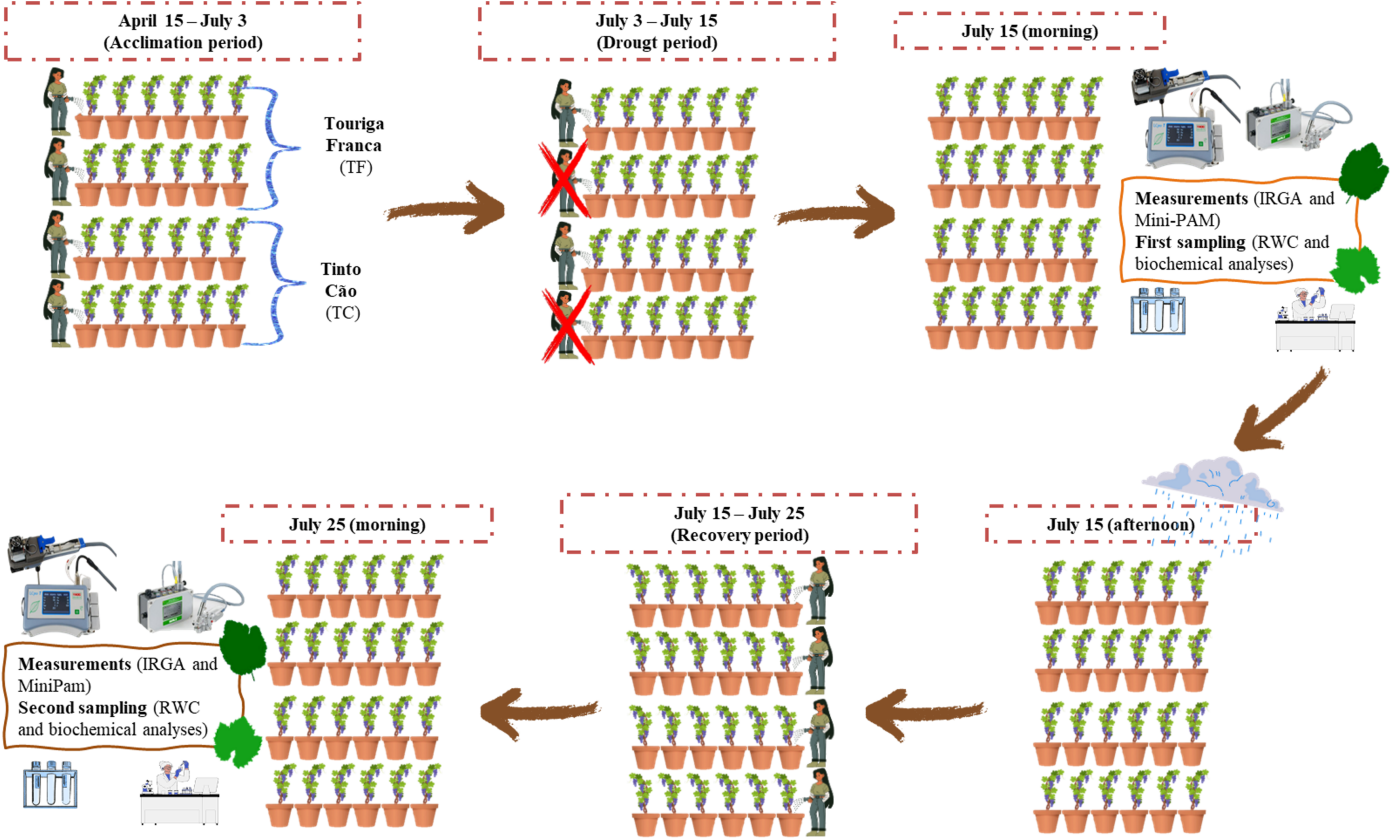
Schematic representation of the experimental design of the pot trial with two grapevine cultivars (Touriga Franca and Tinto Cão) subjected to different water regimes (control and water deficit). The main dates of the application of the water regimes (drought and well-watered conditions) are indicated, along with the timing of physiological measurements (IRGA and Mini-PAM) and the collection of plant material for the determination of relative water content (RWC) and biochemical analysis.

The drought period was interrupted on July 15 due to the rainfall that occurred in the afternoon. From this date onwards, all plants were irrigated to field capacity, thus initiating the recovery phase, during which they were maintained under well-watered conditions. This phase continued until July 25, when the second set of physiological measurements was conducted and the biological material was collected (**Figure 1**).

### Leaf gas exchange and chlorophyll *a* fluorescence

Physiological analyses were performed during the experimental period, comprising measurements of leaf gas exchange and chlorophyll *a* fluorescence. These measurements were conducted exclusively in the morning (10:00–11:30 h) on July 15 (end of the drought period) and July 25 (after the recovery phase, under well-watered conditions). Leaf gas exchange parameters were measured using an open-system infrared gas analyzer (IRGA, model LCpro+, ADC Bioscientific Ltd., Hoddesdon, UK). Measurements were taken on fully expanded, sun-exposed leaves. The following parameters were determined: net CO_2_ assimilation rate (*A*), stomatal conductance (*g_s_*), transpiration rate (*E*), and the ratio of intercellular to ambient CO_2_ concentration (*C_i_/C_a_*), calculated according to the equations of Von Caemmerer and Farquhar (1981). The intrinsic water use efficiency (WUE) was estimated as the ratio between *A* and *g_s_* (*A/g_s_*).

Chlorophyll *a* fluorescence was measured on the same leaves and at the same times as gas exchange parameters, using a pulse-amplitude-modulated fluorometer (Mini-PAM, Walz, Effeltrich, Germany), following the protocol adapted from Bernardo et al. (2021). Leaves were first evaluated under ambient light conditions and subsequently dark-adapted for approximately 30 minutes using opaque leaf clips (DLC-8, Walz) to allow the determination of both dark- and light-adapted parameters (Genty et al., 1989). From these measurements, several indicators of photosystem II (PSII) activity were obtained: maximum quantum efficiency (*F_v_*/*F_m_*), effective quantum yield of PSII under light (*ΦPSII* = (*F_m_′ − F_s_*)/*F_m_*′), photochemical quenching (*qP* = (*F_m_′* − *F_s_*)/(*F_m_′ − F_0_′*)), and non-photochemical quenching (*NPQ* = (*F_m_ − F_m_′*)/*F_m_*). The apparent electron transport rate (*ETR*) was estimated as *ETR* = *ΦPSII* × *PPFD* × 0.5 × 0.84, where PPFD is the photosynthetic photon flux density incident on the leaf, 0.5 accounts for the equal energy distribution between photosystems I and II, and 0.84 corresponds to the average leaf absorptance of C3 plants (Bilger and Schreiber, 1986).

### Relative water content

To assess the water status of the plants, one fully expanded and healthy leaf was collected from each of the six pots per irrigation regime, within each cultivar, on two separate dates (15 and 25 July 2024) during the morning, coinciding with the physiological measurements. Each leaf was immediately weighed on an analytical balance (± 0.001 g) to record its fresh weight (FW). Leaves were then hydrated by immersing their petioles in distilled water at 4 °C for 24 hours in a dark environment to restore cellular turgor. After hydration, excess surface water was gently removed with absorbent paper, and turgid weight (TW) was recorded. Finally, leaves were dried in a ventilated oven at 65 °C for 48 hours, or until a constant weight was achieved, to determine dry weight (DW). Relative water content (RWC, %) was then calculated using the formula: RWC = ((FW-DW)/(TW-DW)) * 100.

### Leaf biochemistry

On both sampling dates (15 and 25 July), during the morning period, one fully expanded and healthy leaf was collected from each pot, resulting in six leaves per cultivar and irrigation regime. Immediately after collection, the samples were frozen in liquid nitrogen in the field and subsequently transported to the laboratory. In the laboratory, the leaves were ground using liquid nitrogen and stored at −80 °C until further analyses were performed. All results were expressed on a fresh weight basis.

#### Photosynthetic pigments

To quantify photosynthetic pigments, 10 mg of fresh leaf tissue, previously macerated in liquid nitrogen, were mixed with 4 mL of 80% (v/v) acetone. The extract was vortexed and sonicated for 5 minutes to enhance pigment extraction, followed by centrifugation at 4,000 rpm for 10 minutes at 4 °C. After centrifugation, absorbance of the supernatant was measured at 663, 645, and 470 nm using a microplate spectrophotometer. Because of the light and temperature sensitivity of these pigments, all extraction and quantification steps were performed on ice and protected from direct light. The concentrations of chlorophyll *a* (Chl *a*), chlorophyll *b* (Chl *b*), total chlorophyll (Chl *a*+*b*), and total carotenoids (Car) were calculated according to Arnon (1949) and Lichtenthaler (1987). Results were expressed as micrograms per gram of fresh weight (μg·g^−1^ FW).

#### Phenolic compounds and antioxidant activity

To evaluate the secondary metabolites and antioxidant potential of the leaves, methanolic extracts were prepared at a concentration of 4 mg·mL^−1^, which were used for subsequent biochemical analyses.

Total phenolic content was quantified using the Folin–Ciocalteu colorimetric assay, with absorbance measured at 750 nm, following the procedure described by Rodrigues et al. (2015). Results were expressed as milligrams of gallic acid equivalents per gram of fresh weight (mg GAE·g^−1^ FW).

Flavonoid concentration was determined by the aluminium chloride (AlCl_3_) complex formation method, recording absorbance at 510 nm, as outlined by Rodrigues et al. (2015). Data were expressed as milligrams of catechin equivalents per gram of fresh weight (mg CAE·g^−1^ FW).

The antioxidant capacity was assessed using the Trolox Equivalent Antioxidant Capacity (TEAC) assay, based on the scavenging activity against the ABTS^+^ radical. Absorbance was read at 734 nm, and results were expressed as milligrams of Trolox equivalents per gram of fresh weight (mg TE·g^−1^ FW).

#### Soluble proteins

Soluble proteins were extracted using a phosphate-based buffer (pH 7.5) supplemented with EDTA, PMSF, and polyvinylpyrrolidone (PVP). Ten milligrams of leaf tissue were homogenized in 200 µL of the extraction solution and centrifuged at 12,000 rpm for 30 minutes at 4 °C. Protein content was quantified using the Bradford reagent (Bradford, 1976), with absorbance measured at 595 nm. Bovine serum albumin (BSA) was used to construct the standard curve, and results were expressed as mg BSA equivalents per gram of fresh weight (mg BSAE g^−1^ FW).

#### Soluble sugars

To quantify total soluble sugars, 10 mg of fresh leaf tissue were extracted in 5 mL of 80% ethanol and heated at 80 °C for 1 hour. After cooling, 100 µL of the extract was mixed with 1.5 mL of anthrone reagent and incubated at 100 °C for 10 minutes. Absorbance was measured at 625 nm (Leyva et al., 2008). A glucose standard curve was used for quantification, and results were expressed as µg·g^−1^ fresh weight (FW).

### Statistical analysis

Data were organised in Microsoft Excel and analysed using SPSS 29.0 software (SPSS Software, Chicago, IL, USA). Each pot was considered an independent biological replicate (n = 6 per treatment). Data were tested for normality and homogeneity of variance prior to analysis and analyzed separately for each sampling date. Differences between irrigation regimes and cultivars were evaluated using one-way ANOVA followed by Tukey’s HSD *post hoc* test. Statistical significance was considered at *p* < 0.05, with significance levels indicated as *p* < 0.05 (*), *p* < 0.01 (**), and *p* < 0.001 (***), while non-significant differences were denoted as n.s. In addition, Pearson correlation analysis was performed among all physiological and biochemical variables, and the results were visualized using a heatmap to explore relationships among parameters and cultivar-specific response patterns.

## Results and discussion

### Meteorological conditions

**Figure 2:**
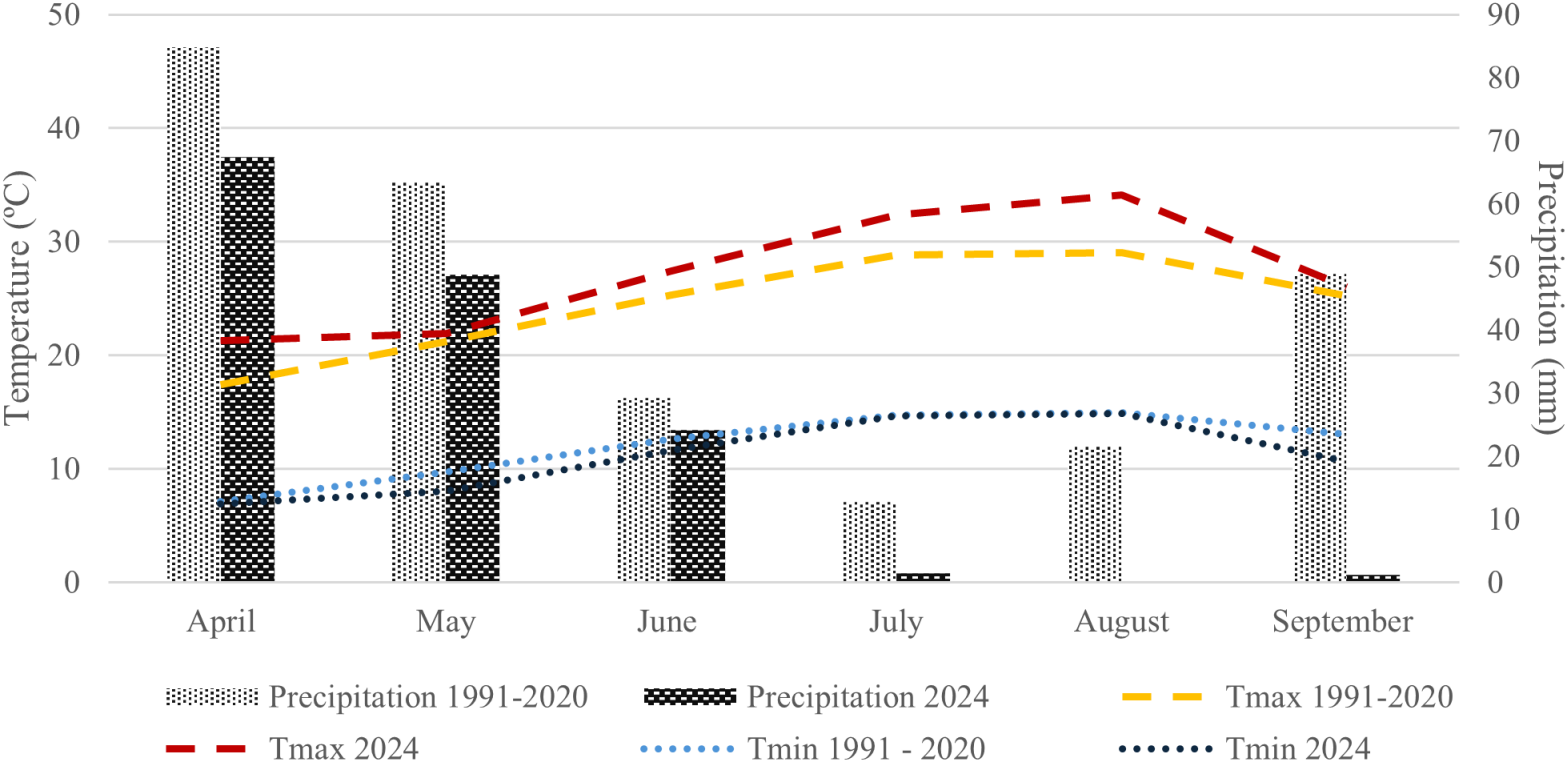
Monthly comparison between historical averages (1991–2020) and recorded values in 2024 for maximum temperature, minimum temperature, and maximum precipitation. Historical average data were obtained from the Portuguese Institute for Sea and Atmosphere (IPMA), while 2024 data were collected from the Weather Underground platform (local weather station – Granja II, Vila Real).

Meteorological conditions prevailing during the experimental period, including precipitation, minimum and maximum temperatures, together with the long-term climatic averages (1991–2020), are presented in **Figure 2**. The Douro Demarcated Region and its sub-regions are known for their highly complex topography and Mediterranean climate (Blanco-Ward et al., 2017), characterized by intense summer water deficit stress (Jones and Alves, 2012). The regional climate is typically Mediterranean-like, classified as warm-temperate with hot and dry summers, and moderate precipitation concentrated mainly in winter, while summer rainfall remains very low (Kottek et al., 2006).

In 2024, climatic conditions diverged substantially from the long-term averages. Maximum temperatures were consistently higher than the 1991–2020 means across all months evaluated, the most pronounced deviations observed in April, July and August., indicating earlier and more intense heat events. Conversely, minimum temperatures remained generally below the historical averages, particularly in May and September, resulting in an increased daily thermal amplitude throughout the study period. Precipitation also differed markedly from the historical regime. A significant reduction in rainfall was recorded across nearly the entire experimental period, with August standing out as a completely dry month. September experienced precipitation above its climatological average, indicating a partial relief from the summer drought. These deviations highlight an intensification of summer aridity followed by a late-summer recovery relative to the 1991–2020 pattern. During July, the month in which physiological measurements were conducted, weather conditions further reflected this anomalous pattern (**Table 1**). Between 3 and 15 July, corresponding to the imposed drought phase, maximum temperatures ranged from 24.0 to 36.6 °C, with one day exceeding 35 °C (4 July) and no precipitation recorded, confirming a period of sustained atmospheric dryness. During the subsequent recovery phase (15–25 July), maximum temperatures fluctuated between 26.5 and 40.2 °C, with four days above 35 °C (22, 23, 24 and 25 July), coinciding with the most intense heat events of the month. Rainfall occurred only once, on 15 July (1.0 mm), representing the sole interruption to otherwise persistently dry summer conditions. These short-term meteorological conditions, characterised by episodic intense heat and minimal rainfall, are consistent with the broader regional trend of pronounced summer aridity and help contextualise the environmental constraints experienced by the vines during the experiment. Overall, these climatic anomalies, combining higher maximum temperatures, lower minimum temperatures, and reduced precipitation, emphasize the vulnerability of viticulture in the Douro region and reinforce the necessity of implementing appropriate adaptation strategies to cope with increasingly variable environmental conditions.

**Table 1:** Daily records of maximum temperature, minimum temperature, and precipitation between July 3rd and 25th, 2024, were obtained directly from the meteorological station of the University of Trás-os-Montes and Alto Douro (UTAD).

	Date	T max (°C)	T min (°C)	Precipitation (mm)
Drougt period	3 July	34.2	15.9	0
	4 July	36.6	15.8	0
	5 July	36.1	17.2	0
	6 July	27.0	12.8	0
	7 July	27.5	9.1	0
	8 July	29.3	9.2	0
	9 July	24.0	15.0	0
	10 July	29.7	17.5	0
	11 July	28.8	16.9	0
	12 July	30.5	12.6	0
	13 July	30.2	12.3	0
	14 July	27.4	10.1	0
Recovery period	15 July	25.2	14.7	1.00
	16 July	28.4	9.2	0
	17 July	33.8	13.5	0
	18 July	34.3	12.3	0
	19 July	34.0	14.0	0
	20 July	26.5	15.4	0
	21 July	31.5	11.8	0
	22 July	35.6	14.4	0
	23 July	40.2	17.2	0
	24 July	39.7	18.3	0
	25 July	37.3	17.6	0

### Divergent responses of cultivars to leaf gas exchange under water deficit

Leaf gas exchange parameters are presented in **Table 2**, and as expected, during the water deficit period (up to 15 July), both cultivars showed significant reductions in *A* and *g_s_* compared with continuously irrigated plants (*p* < 0.001). Stomatal conductance (*g_s_*) was interpreted as a physiological indicator of stomatal regulation in response to water deficit rather than as a direct measure of plant water status, since *g_s_* reflects cultivar-specific hydraulic strategies such as isohydric or anisohydric behavior. Touriga Franca (TF) plants in water-deficit exhibited a reduction in *g_s_* of approximately 44% relative to continuously irrigated plants, while net CO_2_ assimilation decreased by 33%. However, *A/g_s_* increased by 39% in these plants, suggesting a partial stomatal closure strategy that allows maintenance of some carbon assimilation during the early stages of drought while limiting excessive water loss (Chaves et al., 2010). In contrast, Tinto Cão (TC) displayed a more conservative response, with a pronounced reduction in *g_s_* (≈77%) and *A* (≈56%) under water deficit compared with continuously irrigated plants. The Ci/Ca ratio decreased slightly, reflecting the tendency for stomatal closure characteristic of an isohydric behaviour, which favours water conservation and maintenance of plant water status (Chaves et al., 2010; Hochberg et al., 2018), however, this reduction was not statistically significant. Despite these reductions, *A/g_s_* increased by 29%, confirming the capacity of TC to maintain physiological functionality even under water deficit stress (Hochberg et al., 2013).

**Table 2:** Leaf gas exchange parameters, namely transpiration rate (*E*), stomatal conductance (*g_s_*), net CO_2_ assimilation rate (*A*), intrinsic water use efficiency (*A/g_s_*) and intercellular/atmospheric CO_2_ concentration ratio (*Ci/Ca*), measured on 15 and 25 July 2024 (after the drought and recovery periods, respectively), in different grapevine cultivars with different hydric regimes (N = 6 pots per treatment).

Cultivar/Irrigation regime	*E*	*g_s_*	*A*	*A/g_s_*	*Ci/Ca*
	(mmol·m^-2^·s^-1^)	(mmol·m^-2^·s^-1^)	(µmol· m^-2^·s^-1^)	(µmol·mol^-1^)	
15 July (after the drought period)					
TF – I	3.83 ± 0.396 a	279.3 ± 13.3 c	12.5 ± 1.72 b	44.3 ± 3.03 a	0.765 ± 0.027
TF – NI	4.78 ± 0.458 a	152.3 ± 13.3 b	8.43 ± 1.67 a	61.7 ± 3.01 b	0.708 ± 0.029
TC – I	7.19 ± 0.374 b	264.0 ± 12.5 c	12.0 ± 1.54 b	43.1 ± 2.80 a	0.728 ± 0.048
TC – NI	3.52 ± 0.884 a	61.6 ± 22.8 a	5.31 ± 1.20 a	55.5 ± 6.09 b	0.691 ± 0.065
*Significance*	***	***	***	***	*n.s.*
25 July (after the recovery period)					
TF – PI	3.85 ± 0.469	233.0 ± 30.0 b	10.2 ± 1.20	55.3 ± 7.95 a	0.722 ± 0.064
TF – PNI	3.30 ± 0.655	165.3 ± 25.3 a	10.1 ± 1.51	68.4 ± 8.11 a	0.712 ± 0.068
TC – PI	3.08 ± 0.327	117.8 ± 19.0 a	8.67 ± 1.15	68.8 ± 6.48 a	0.722 ± 0.083
TC – PNI	3.61 ± 0.551	149.3 ± 17.1 a	9.07 ± 1.26	56.0 ± 3.93 a	0.734 ± 0.035
*Significance*	*n.s.*	***	*n.s.*	*	*n.s.*

I, irrigated; NI, non-irrigated; PI, previously irrigated; PNI, previously non-irrigated.

Data are expressed as mean ± standard deviation. Statistical analysis was performed using one-way ANOVA followed by Tukey’s *post hoc* test. Different letters indicate significant differences between treatments (cultivars and irrigation regimes). Significance levels for the analysis of variance (ANOVA): *p* < 0.05 “*”, *p* < 0.01 “**”, *p* < 0.001 “***”, not significant – “n.s.” (*p* > 0.05).

Following full rehydration, non-irrigated plants showed partial recovery, with gas exchange values on 25 July approaching those of continuously irrigated plants. Specifically, Touriga Franca recovered CO_2_ assimilation, reaching values similar to irrigated plants after the recovery period. Interestingly, Tinto Cão previously subjected to water deficit exhibited slightly higher A values than continuously irrigated plants, although these differences were not statistically significant, suggesting a possible post-irrigation compensatory effect.

A comparison between the two cultivars highlights their inherent physiological differences: under both irrigation and water-deficit conditions, TF generally maintained higher *g_s_* and *A* than TC, reflecting a less conservative water-use strategy. Conversely, TC exhibited lower *g_s_* and *A* but tended to achieve higher water use efficiency under stress, consistent with a more isohydric behavior. This inter-cultivar comparison indicates that TF prioritizes carbon assimilation even under moderate stress, whereas TC adopts stricter stomatal control to conserve water, revealing contrasting adaptive strategies to environmental fluctuations.

Overall, the gas exchange analysis indicates that TF exhibits a more balanced response to water deficit: it maintains moderate photosynthetic activity and high water use efficiency during drought, and recovers quickly after rewatering, displaying strategies similar to anisohydric genotypes (Chaves et al., 2010; Hochberg et al., 2013). In contrast, TC adopts a more conservative stomatal closure strategy, resulting in a pronounced reduction in photosynthesis to conserve water, characteristic of isohydric behaviour, yet retaining functional resilience after rehydration (Hochberg et al., 2018). These contrasting strategies suggest distinct adaptive approaches: TF shows potential to thrive under controlled irrigation with intermittent drought, whereas the conservative TC may require more frequent irrigation to avoid substantial reductions in photosynthesis, which could significantly affect both plant performance and yield.

Taken together, the combination of physiological parameters provides insight into the contrasting water-management strategies of the two cultivars. The relatively higher *g_s_* and *A* observed in Touriga Franca during water deficit indicate a less conservative stomatal regulation strategy, allowing continued carbon assimilation despite reduced water availability. In contrast, the stronger reduction in *g_s_* in Tinto Cão, combined with the maintenance of higher RWC, suggests tighter stomatal control aimed at conserving plant water status. These patterns are consistent with the contrasting hydraulic strategies often described in grapevine cultivars, where anisohydric genotypes maintain gas exchange at the expense of higher water loss, while more isohydric genotypes prioritize water conservation through earlier stomatal closure.

### Photoprotective strategies to induce cultivar resilience

During the drought period, plants of TF and TC cultivars subjected to water deficit exhibited significant changes in some photophysiological parameters, as presented in **Table 3**. Although maximum quantum efficiency of photosystem II (*F_v_/F_m_*) and effective quantum yield (*ΦPSII*) did not show statistically significant differences between treatments, indicating the absence of irreversible damage to *PSII* (Baker, 2008; Malnoë, 2018), significant reductions in photochemical quenching (*qP*) and increases in non-photochemical quenching (*NPQ*) were observed in water-deficit plants.

**Table 3:** Chlorophyll a fluorescence parameters, namely effective efficiency of PSII (ΦPSII), photochemical quenching (qP), maximum quantum efficiency of photosystem II (Fv/Fm), and non-photochemical quenching (NPQ), measured on 15 and 25 July 2024 (after the drought and recovery periods, respectively), in different grapevine cultivars with different hydric regimes (N = 6 pots per treatment).

Cultivar/Irrigation Regime	*F_v_/F_m_*	*ΦPSII*	*qP*	*NPQ*
15 July (after the drought period)				
TF – I	0.710 ± 0.068	0.290 ± 0.090	0.681 ± 0.040 b	2.50 ± 0.932 a
TF – NI	0.735 ± 0.085	0.197 ± 0.043	0.671 ± 0.039 b	4.91 ± 0.779 b
TC – I	0.809 ± 0.070	0.219 ± 0.098	0.672 ± 0.052 b	2.19 ± 0.529 a
TC – NI	0.822 ± 0.076	0.172 ± 0.069	0.376 ± 0.061 a	3.07 ± 0.726 a
*Significance*	*n.s.*	*n.s.*	***	***
25 July (after the recovery period)				
TF – PI	0.786 ± 0.068	0.093 ± 0.018	0.369 ± 0.059	8.25 ± 0.696
TF – PNI	0.832 ± 0.064	0.121 ± 0.025	0.433 ± 0.036	8.04 ± 0.834
TC – PI	0.796 ± 0.078	0.120 ± 0.015	0.366 ± 0.066	8.00 ± 0.865
TC – PNI	0.767 ± 0.051	0.125 ± 0.025	0.353 ± 0.055	6.97 ± 0.383
*Significance*	*n.s.*	*n.s.*	*n.s.*	*n.s.*

I, irrigated; NI, non-irrigated; PI, previously irrigated; PNI, previously non-irrigated.

Data are expressed as mean ± standard deviation. Statistical analysis was performed using one-way ANOVA followed by Tukey’s *post hoc* test. Different letters indicate significant differences between treatments (cultivars and irrigation regimes). Significance levels for ANOVA: p < 0.001 “***”, not significant – “n.s.” (p > 0.05).

In fact, the decrease in *qP* in non-irrigated plants, particularly in TC, which showed a reduction of approximately 44% compared with irrigated plants, suggests a limitation in reaction centre opening and electron transport, reflecting reduced photosynthetic activity under water deficit (Baker, 2008; Tikkanen et al., 2014), which is in accordance with the *A* observed in the **Table 2**. The increase in *NPQ* in water-deficit plants was more pronounced in TF, which exhibited a 96% increase relative to the irrigated control, highlighting the activation of thermal dissipation mechanisms as a protection against excess light energy and stress (Murchie and Lawson, 2013; Ripoll et al., 2016).

After the recovery period, photophysiological parameters of previously water-deficit plants approached values observed in continuously irrigated plants. Indeed, no statistically significant differences were recorded on 25 July for any parameters. Interestingly, TC showed similar *qP* values between irrigated and non-irrigated plants after recovery, suggesting a possible post-irrigation compensatory effect, where the plant optimises the use of light energy for photosynthesis (Gonçalves et al., 2007).

A direct comparison between cultivars reveals that, under water-deficit conditions, TF tends to activate stronger thermal dissipation (higher *NPQ*), whereas TC exhibits a more pronounced limitation in *PSII* reaction center opening (lower *qP*). This suggests that TF relies more on energy dissipation to protect photosystems, while TC restricts photochemistry to reduce stress on the photosynthetic machinery. These inter-cultivar differences highlight distinct photoprotective strategies, consistent with their contrasting stomatal behavior and water-use responses.

These results indicate that during water deficit, vines activate effective photoprotective mechanisms, reflected by increased *NPQ* in TF and reduced *PSII* reaction centre opening (*qP*) in TC, thereby preventing PSII photoinhibition, as evidenced by the stability of *Fv*/*Fm*. The rapid recovery after rehydration confirms the resilience of both cultivars under moderate drought, supporting the existence of distinct adaptive strategies to cope with water deficit (Adams et al., 2008; Hochberg et al., 2018).

### Higher water retention capacity of TC cultivar under water deficit conditions

Relative water content (RWC) is a widely used indicator to assess plant water status, reflecting the amount of water present in leaf tissues at the time of sampling, expressed as a percentage of the water that the leaf could retain at full turgor (Barrs and Weatherley, 1962). This parameter allows evaluation of the plant’s functional capacity to maintain tissue hydration during periods of water deficit and is considered one of the most direct physiological indicators of cellular water availability.

**Figure 3:**
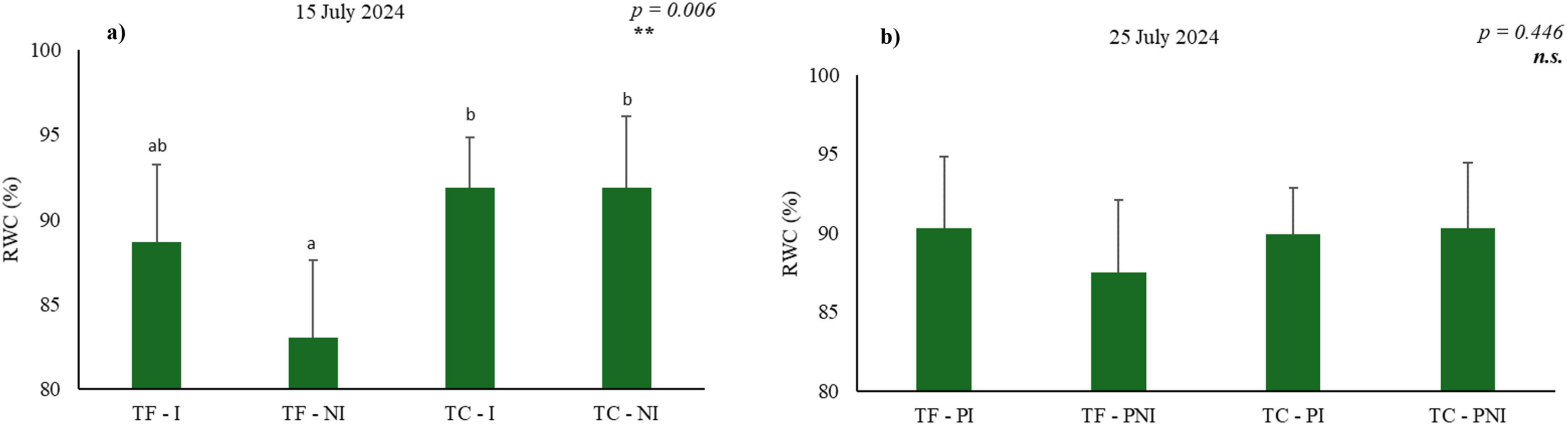
Relative water content (RWC, %) measured on 15 July 2024, at the end of the drought period **(a)**, and on 25 July 2024, at the end of the recovery period **(b)**, in different grapevine cultivars with different hydric regimes (N=6 pots per treatment). Data are expressed as mean ± standard deviation. Statistical analysis was performed using one-way ANOVA followed by Tukey’s post hoc test. Different lowercase letters indicate significant differences between treatments (cultivars and irrigation regimes). Significance levels for ANOVA: p < 0.01 “**”, not significant – “n.s.” (p > 0.05). I, irrigated; NI, non-irrigated; PI, previously irrigated; PNI, previously non-irrigated.

On 15 July 2024 (**Figure 3a**), after a drought period, significant differences were observed between treatments (*p* = 0.006). Under water deficit, TF exhibited an RWC of 83.0%, representing a reduction of approximately 6.4% compared with irrigated plants (88.7%). This decrease indicates a moderate response to water deficit, close to the 75–85% threshold at which photosynthesis may begin to be negatively affected (Peñuelas et al., 1993; Escalona et al., 2000). In contrast, TC maintained remarkable water stability, with RWC values above 91% under both irrigation regimes, reflecting optimal hydration and demonstrating a greater capacity of this cultivar to preserve leaf water status even under drought conditions.

After the recovery period, on 25 July (**Figure 3b**), no statistically significant differences in RWC were observed among treatments (*p* = 0.446), with all plants showing high and similar hydration levels. Nevertheless, TF plants previously subjected to water deficit maintained slightly lower values (87.5%) compared with continuously irrigated plants (90.3%), suggesting partial recovery of leaf hydration. In TC, RWC remained high and stable, around 90%, regardless of irrigation regime, reinforcing its resilience and efficiency in maintaining leaf water status.

It is important to note that, according to several authors (Peñuelas et al., 1993; Bongi and Palliotti, 1994; Escalona et al., 2000), RWC values below 75–80% significantly impair photosynthetic metabolism. Therefore, the data from this study suggest that neither cultivar reached critical dehydration levels, although TF exhibited greater sensitivity to water deficit, indicating mild stress in non-irrigated plants. Conversely, TC showed higher physiological stability, maintaining RWC at levels compatible with optimal photosynthetic function under both irrigation regimes.

### Changes in photosynthetic pigments in TC and TF under different irrigation regimes: stability versus recovery

Based on photosynthetic pigment data (**Table 4**), no statistically significant differences were observed between irrigation regimes on 15 July, corresponding to the end of the drought period, for chlorophyll a (Chl a), chlorophyll b (Chl b), total chlorophyll (Chl a + b), and carotenoids (Car). However, in TF, plants subjected to water deficit showed a slight reduction (7.5%) in Chl a compared with irrigated plants, while Car content increased by approximately 20%. In contrast, TC maintained Chl a almost unchanged between irrigated and non-irrigated plants (variation of only 2.3%), and Car levels varied by just 6.3%, suggesting greater stability of photosynthetic pigments under stress conditions.

**Table 4:** Photosynthetic pigment content (chlorophyll a – Chl a, chlorophyll b – Chl b, total chlorophyll – Chl a + b, and carotenoids – Car) in leaves of Touriga Franca and Tinto Cão measured on 15 and 25 July 2024 (after the drought and recovery periods, respectively), in different grapevine cultivars with different hydric regimes (N = 6 pots per treatment).

Cultivar/Irrigation Regime	Chl *a*	Chl *b*	Chl *a + b*	Car
	(mg·g^-1^)	(mg·g^-1^)	(mg·g^-1^)	(mg·g^-1^)
15 July (after the drought period)				
TF – I	1.73 ± 0.124	1.71 ± 0.164	3.44 ± 0.272	0.35 ± 0.057
TF – NI	1.60 ± 0.235	1.72 ± 0.205	3.32 ± 0.432	0.42 ± 0.108
TC – I	1.76 ± 0.264	1.71 ± 0.314	3.47 ± 0.574	0.32 ± 0.047
TC – NI	1.72 ± 0.261	1.63 ± 0.181	3.34 ± 0.430	0.34 ± 0.095
*Significance*	*n.s.*	*n.s.*	*n.s.*	*n.s.*
25 July (after the recovery period)				
TF – PI	1.44 ± 0.259 a	1.71 ± 0.324	3.15 ± 0.569	0.256 ± 0.073 a
TF - PNI	1.65 ± 0.148 ab	1.79± 0.329	3.44 ± 0.472	0.316 ± 0.071 a
TC - PI	1.73 ± 0.115 b	1.72 ± 0.137	3.45 ± 0.234	0.330 ± 0.055 a
TC - PNI	1.42 ± 0.108 a	1.59 ± 0.154	3.02 ± 0.232	0.230 ± 0.056 a
*Significance*	*	*n.s.*	*n.s.*	*

I, irrigated; NI, non-irrigated; PI, previously irrigated; PNI, previously non-irrigated.

Data are expressed as mean ± standard deviation. Statistical analysis was performed using one-way ANOVA followed by Tukey’s *post hoc* test. Different letters indicate significant differences between treatments (cultivars and irrigation regimes). Significance levels for ANOVA: p < 0.05 “*”, not significant – “n.s.” (p > 0.05).

After the recovery period on 25 July, significant differences were observed in Chl a and Car content. In TF, previously water-deficit plants exhibited Chl a level 14.6% higher than continuously irrigated plants. In TC, a 17.9% reduction in Chl a was observed under water deficit compared with irrigated plants. Simultaneously, Car content in TC decreased by 30.3% under water deficit, although differences were not statistically significant. This pattern is consistent with previous studies indicating that the TC cultivar adopts a distinct adaptive strategy, characterized by maintaining lower levels of photosynthetic pigments under stress. In our results, however, this reduction was not pronounced enough to confirm effects on excessive light absorption or on the production of reactive oxygen species (ROS) (Moutinho-Pereira et al., 2007; Wang et al., 2022). This preventive strategy, which includes lighter-colored leaves and higher reflectance, is considered an effective mechanism to minimize photo-oxidative damage in high-light and high-temperature environments, such as the Douro region during summer (Moutinho-Pereira et al., 2007; Palliotti et al., 2015). Conversely, TF may exhibit greater plasticity in its photosynthetic pigment composition, adapting to water deficit by maintaining relative levels of chlorophyll and carotenoids. This behaviour suggests a certain resilience of the photosynthetic apparatus, similar to the findings of Zulini et al. (2007), who reported pigment stability and a significant correlation between photosynthetic efficiency (F_v_/F_m_) and chlorophyll content under prolonged water stress.

When comparing the two cultivars, TF tends to maintain slightly higher levels of chlorophyll a and b than TC, both during the water deficit period and after recovery. Conversely, TC shows greater stability in carotenoid content, even under water stress, suggesting that this cultivar adopts a more preventive strategy, thereby reducing the risk of photoinhibition and oxidative damage. In contrast, TF exhibits greater plasticity in pigment composition, reflecting a faster adjustment and recovery capacity following stress alleviation.

Furthermore, the relationship between chlorophyll and carotenoid levels underscores the protective role of carotenoids against pigment degradation and photo-oxidative damage (Smirnoff, 1993; Moutinho-Pereira et al., 2007). Carotenoids play a crucial role in dissipating excess energy as heat (non-photochemical quenching), protecting the photosynthetic apparatus from irreversible damage (Moutinho-Pereira et al., 2007). Thus, the greater pigment stability observed in TC may reflect a preventive strategy, while the higher variability and recovery of pigments in TF indicate a more reactive, repair-oriented response following stress.

The relative stability of photosynthetic pigments and carotenoids in both cultivars further supports the maintenance of photosystem functionality under moderate water deficit, contributing to the overall resilience of the photosynthetic apparatus.

### Dynamics of leaf biochemical compounds under different irrigation regimes

#### Transient increase in total phenolics in TC under water deficit

Phenolic compounds are abundant secondary metabolites in plants, involved in pigmentation, growth, and protection against biotic and abiotic stress, including water deficit and ultraviolet radiation (Naczk and Shahidi, 2006; Król et al., 2014). In grapevine, they accumulate mainly in berries, skins, and seeds (Waterhouse, 2002) and are influenced by factors such as light exposure and soil water availability (Singleton, 1988; Matthews et al., 1990). High phenolic content in fruits generally indicates the absence of severe stress, as under extreme conditions these compounds may be redirected to leaves (Matthews et al., 1990). Additionally, phenolics are well known for their antioxidant properties, with beneficial effects on human health (Achkar et al., 2013; Dinis et al., 2016).

Analysis of total phenolic content in leaves of the two cultivars revealed significant differences between irrigation regimes, particularly after the drought period (**Table 5**). On 15 July, TC plants under water deficit exhibited total phenolic content 26.2% higher than irrigated plants. Similarly, TF showed a 5.5% increase in total phenolics in response to water restriction. This more pronounced accumulation of phenolic compounds in TC during stress suggests a stronger physiological response to water deficit, likely related to an increased need for antioxidant protection against ROS (Elavarthi and Martin, 2010; Naikoo et al., 2019).

**Table 5:** Biochemical parameters assessed in the leaves of Touriga Franca (TF) and Tinto Cão (TC) cultivars on 15 and 25 July (after the drought period and following rewatering, respectively), in different grapevine cultivars with different hydric regimes (N = 6 pots per treatment).

Cultivar/Irrigation Regime	Phenols	Flavonoids	Soluble proteins	Soluble sugars	ABTS
	(mg·g^-1^)	(mg·g^-1^)	(mg·g^-1^)	(mg·g^-1^)	(mg·g^-1^)
15 July (after the drought period)					
TF – I	27.2 ± 0.835 a	16.2 ± 4.15	1.10 ± 0.658 a	4.01 ± 0.302 b	0.851 ± 0.063
TF – NI	28.7 ± 0.721 b	18.4 ± 3.56	1.06 ± 0.336 a	3.38 ± 0.283 ab	0.959 ± 0.065
TC – I	26.3 ± 0.867 a	17.4 ± 1.03	1.63 ± 0.461 a	3.14 ± 0.159 a	0.812 ± 0.122
TC – NI	33.2 ± 1.13 c	17.4 ± 3.41	10.2 ± 1.63 b	3.77 ± 0.326 ab	0.889 ± 0.088
*Significance*	***	*n.s.*	***	**	*n.s.*
25 July (after the recovery period)					
TF – PI	23.6 ± 1.14 a	11.5 ± 2.89	1.93 ± 0.990 b	4.56 ± 0.717	0.875 ± 0.084 b
TF – PNI	26.3 ± 0.999 b	12.0 ± 3.56	1.37 ± 0.462 ab	4.39 ± 0.600	0.946 ± 0.078 b
TC – PI	25.3 ± 0.919 b	10.7 ± 1.44	0.367 ± 0.166 a	3.92 ± 0.040	0.663 ± 0.082 a
TC – PNI	25.9 ± 1.09 b	8.34 ± 1.01	1.26 ± 0.585 ab	3.63 ± 0.233	0.870 ± 0.091 b
*Significance*	**	*n.s.*	**	*n.s.*	***

I, irrigated; NI, non-irrigated; PI, previously irrigated; PNI, previously non-irrigated.

Data are expressed as mean ± standard deviation. Statistical analysis was performed using analysis of variance (ANOVA), followed by the Tukey *post hoc* test. Different lowercase letters indicate statistically significant differences between treatments (cultivars and irrigation regimes). ANOVA significance: “***” p<0.001; “**” p<0.01; n.s.– not significant (p>0.05).

At the second sampling date (25 July), after irrigation was restored to all plants, differences in total phenolic content between irrigation regimes were observed (**Table 5**). In TF, plants previously subjected to water deficit showed slightly higher phenolic levels than continuously irrigated plants, in contrast, TC exhibited similar values between treatments, indicating a stable response and efficient recovery of metabolic balance (Dixon and Paiva, 1995; Fini et al., 2011). In summary, TC exhibits a stronger response to water deficit but recovers quickly after rewatering, whereas TF shows a more moderate and prolonged accumulation, highlighting clear differences in metabolic plasticity between the two cultivars.

#### Temporary accumulation and subsequent decline of flavonoids in response to water deficit and recovery

Flavonoids are widely distributed secondary phenolic compounds in plant tissues, playing key roles in protection against abiotic stress, such as water deficit and UV radiation, through scavenging ROS and preserving cellular integrity (Gouot et al., 2019; Liang et al., 2021). These molecules contribute to the plant’s adaptive response by activating specific metabolic pathways that promote the synthesis of various phenolic compounds, including flavonoids (Liang et al., 2021). In grapevine, these compounds are particularly important due to their influence on wine quality. They are also associated with antioxidant properties beneficial to human health, notably in cardiovascular protection (Gutiérrez-Escobar et al., 2021). Flavonoid composition can vary considerably depending on cultivar, environmental conditions, and irrigation regime, with accumulation often stimulated under stress conditions (Gouot et al., 2019).

Analysis of flavonoid content in leaves of TF and TC at two time points, 15 July (after the drought period) and 25 July (after recovery), revealed some variations, although not statistically significant (**Table 5**). At the end of the drought period, TF showed slightly higher flavonoid values under water deficit, suggesting a possible activation of phenolic biosynthetic pathways in response to stress (Liang et al., 2021). In TC, flavonoid levels were similar between treatments, indicating a more stable response in this parameter. Between the two sampling dates, a decrease in flavonoid content was observed in both cultivars, being more pronounced in TC. This reduction after rehydration may indicate a downregulation of defence-related metabolism once stress conditions were alleviated, in line with previous findings describing a transient accumulation of flavonoids under drought stress (Gouot et al., 2019).

Comparatively, TF exhibited a more controlled and gradual response during and after water deficit, suggesting greater adaptive regulation capacity. In contrast, TC showed high post-stress sensitivity, reflected in a marked reduction in flavonoid content, which may compromise leaf antioxidant efficiency at this stage. This dynamic between accumulation and degradation of flavonoids may have implications for both plant physiology and the phenolic profile of grapes, and consequently, wine quality (Rustioni et al., 2012; Gutiérrez-Escobar et al., 2021).

#### TC accumulates soluble proteins under water deficit

Proteins, including stress-related ones, might be upregulated to help mitigate the damage and repair cellular components (Dinis et al., 2024). Proteins could be essential for stress responses, aiding in cellular maintenance and enzymatic activities that mitigate damage caused by environmental stressors (Pereira et al., 2025).

Regarding leaf soluble protein content (**Table 5**), significant differences were observed between irrigation regimes at both sampling dates. On 15 July, after the drought period, the highest protein content was recorded in TC under water deficit, significantly higher than all other treatments. This represents an extreme increase compared to irrigated TC, reflecting a strong physiological response to water deficit, notably through protein accumulation as a protective mechanism (Hong et al., 2000; Król and Weidner, 2017). The synthesis of various types of proteins in grapevine leaves under water deficit has previously been described as an adaptive strategy with a relevant impact on metabolism (Negri et al., 2008).

In TF, soluble protein levels were similar between irrigation regimes, with a slight reduction of 3.6% in water-deficit plants compared to irrigated ones, suggesting a lower sensitivity or a more moderate stress response in this parameter.

At the second sampling date, 25 July, after rehydration of previously water-deficit plants, soluble protein levels became more homogeneous across treatments. TF with irrigation showed 41% higher protein content than TF previously under deficit, reflecting a more efficient recovery of protein metabolic activity following rehydration. In TC, soluble protein content decreased by 71% in irrigated plants compared to those previously under stress, highlighting the importance of protein accumulation in the response to water deficit and its subsequent decline under favorable conditions (Hong et al., 2000).

Overall, TC exhibits a strong but temporary protein accumulation under water deficit, while TF maintains more stable levels, suggesting differences in stress sensitivity and metabolic adjustment between the two cultivars.

#### TF accumulates higher soluble sugar content under continuous irrigation

Soluble sugars (**Table 5**) play a key role in grapevine physiology, serving as a primary source of energy and carbon. They transiently accumulate in leaves, during the day, as photoassimilates, before being translocated to storage or growth organs, such as the berries (Hunter et al., 2017). This transport occurs mainly post-veraison via mass flow in the phloem and is highly dependent on leaf photosynthetic activity (Ollat and Gaudillère, 2000). Under stress conditions, such as water deficit, sugar accumulation in leaves may act as a protective mechanism to maintain cellular homeostasis (Boussadia et al., 2013).

After the drought period, TF and TC leaves showed distinct responses to irrigation. TF plants under irrigation exhibited the highest sugar content, significantly exceeding that of irrigated TC by 27.7%, whereas TF under water deficit had sugar levels 15.7% lower than irrigated TF. In contrast, TC under water deficit accumulated 20% more sugars than irrigated plants, indicating a different strategy of carbohydrate management under drought. These results suggest that TF and TC respond differently to irrigation, TF exhibits a greater capacity for sugar accumulation under irrigation, possibly due to a more efficient metabolism for photoassimilate fixation and translocation, whereas TC shows a less pronounced increase (Ollat and Gaudillère, 2000; Hunter et al., 2017). Additionally, sugar accumulation under water deficit may act as a tolerance mechanism, helping to maintain cellular homeostasis (Boussadia et al., 2013).

By 25 July, following the recovery period, soluble sugar contents were similar between irrigation regimes in both cultivars, with no statistically significant differences. In TF, irrigated and previously non-irrigated plants reached comparable values, indicating a balanced carbohydrate status after rehydration. In TC, sugar levels were slightly higher in irrigated plants, suggesting that water availability may have supported a more efficient photosynthetic recovery. Overall, the absence of significant differences after rewatering indicates a tendency toward stabilization of sugar metabolism, consistent with reports of rapid metabolic adjustment following stress relief (Ollat and Gaudillère, 2000).

#### Antioxidant resilience of TF under water deficit and recovery

The leaf antioxidant capacity of TF and TC grapevine cultivars, assessed using the ABTS method (**Table 5**), exhibited distinct responses over time under different water regimes. On 15 July, following the period of water deficit, no statistically significant differences were observed between irrigated and non-irrigated treatments, suggesting that both cultivars were able to maintain relatively stable antioxidant activity during the early phase of drought. However, the second sampling date (25 July), after rewatering, significant differences in antioxidant activity were observed between irrigation regimes. In both cultivars, non-irrigated plants presented higher ABTS values compared to irrigated ones.

The resilience observed in the TF cultivar may be associated with a more efficient metabolism in the production and maintenance of antioxidant compounds, notably phenolics and flavonoids, which have been identified as the primary agents responsible for neutralizing free radicals in plant tissues (Burns et al., 2000; Yildirim et al., 2005; Rockenbach et al., 2011). These compounds act as electron or hydrogen donors, stabilizing reactive intermediates (Yilmaz and Toledo, 2006), and their abundance is frequently correlated with high ABTS-measured antioxidant activity (Da Silva et al., 1991; Wang and Lin, 2000). Furthermore, the structural profile of phenolic compounds, particularly the presence of hydroxyl groups at specific positions, significantly contributes to antioxidant efficacy (Zainol et al., 2003; Rosicka-Kaczmarek, 2004).

The higher ABTS values in TF suggest that this cultivar is better equipped to neutralize ROS and maintain cellular integrity under stress, consistent with the observed accumulation of phenolic compounds and flavonoids during water deficit.

Overall, the results indicate that the maintenance of high antioxidant capacity, particularly evident in the TF cultivar, may provide adaptive advantages under water deficit conditions by protecting against oxidative damage and preserving cellular integrity.

From a viticultural perspective, the contrasting physiological responses observed between the two cultivars may have important implications for vineyard management under increasing water scarcity. The capacity of Touriga Franca to maintain relatively higher stomatal conductance and photosynthetic activity during moderate drought suggests that this cultivar may be better suited to deficit irrigation strategies, where controlled water limitation is applied to optimize water use while maintaining physiological activity. In contrast, the stronger stomatal regulation and higher leaf water retention observed in Tinto Cão indicate a more conservative water-use strategy, which may confer advantages under severe drought conditions by limiting plant dehydration, although potentially at the cost of reduced carbon assimilation.

These contrasting physiological behaviors highlight the importance of cultivar selection as an adaptive strategy for viticulture in Mediterranean regions, where increasing temperatures and reduced precipitation are expected to intensify water stress during the growing season.

### Correlation analysis reveals coordinated physiological and biochemical responses to water deficit

**Figure 4:**
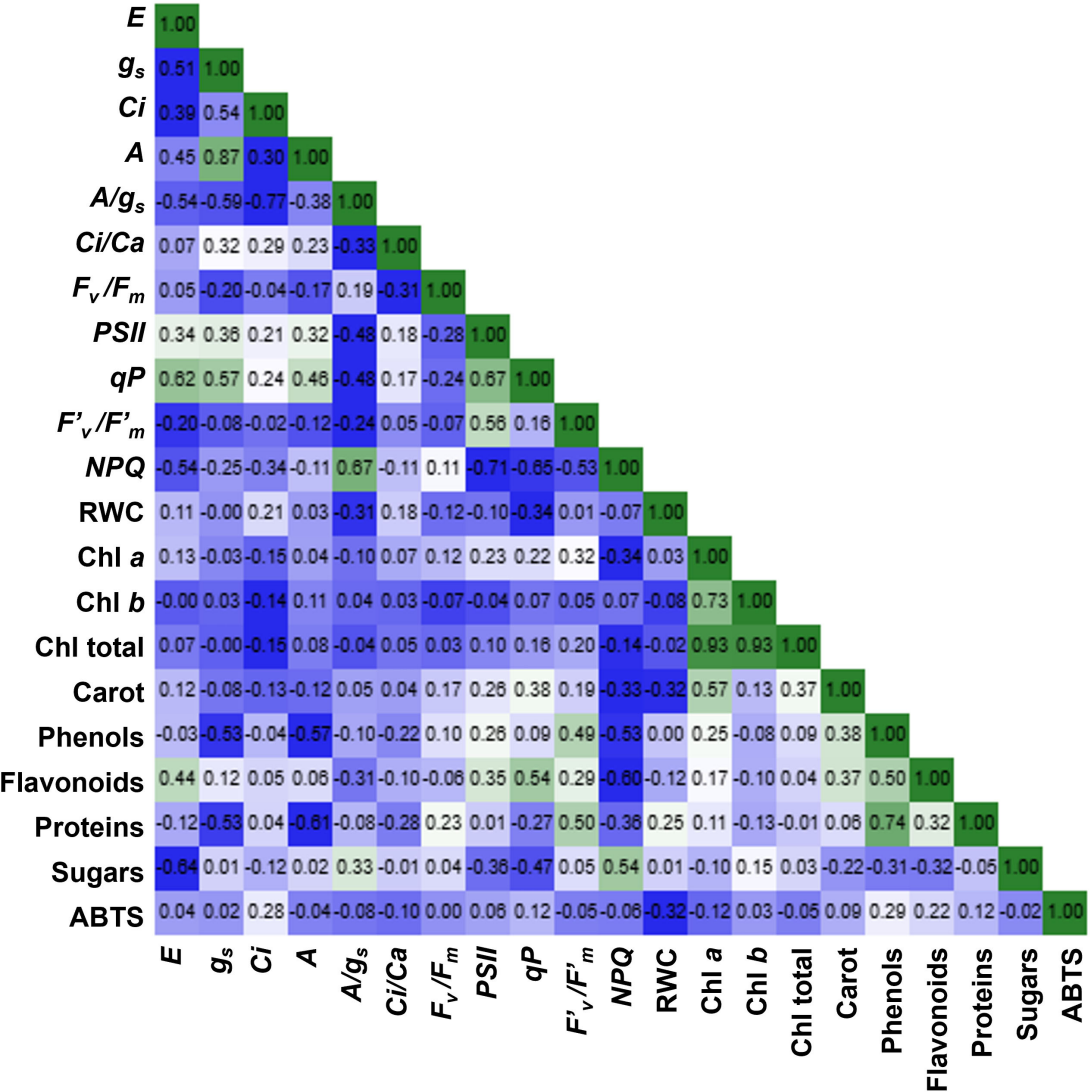
Pearson correlation heatmap showing the relationships among physiological and biochemical parameters measured in grapevine leaves. The colour scale represents the strength and direction of correlations, with positive correlations shown in green and negative correlations in blue. Values inside the squares correspond to Pearson correlation coefficients (r). Physiological variables include transpiration rate (*E*), stomatal conductance (*g_s_*), intercellular CO_2_ concentration (*Ci*), net photosynthetic rate (*A*), intrinsic water use efficiency (*A/g_s_*), and *Ci/Ca* ratio, together with chlorophyll fluorescence parameters (*F_v_/F_m_*, *ΦPSII*, *qP*, *F′_v_/F′_m_*, *NPQ*). Biochemical parameters include relative water content (RWC), chlorophyll *a* (Chl *a*), chlorophyll *b* (Chl *b*), total chlorophyll (Chl total), carotenoids (Carot), phenolic compounds (Phenols), flavonoids, soluble proteins, soluble sugars, and antioxidant activity (ABTS).

A Pearson correlation heatmap was generated to explore the relationships among physiological and biochemical parameters measured in the study (**Figure 4**). The analysis revealed several clear functional associations among variables related to grapevine drought responses.

Gas exchange parameters showed strong positive correlations, particularly between *A* and *g_s_* (r = 0.87), indicating that photosynthetic performance was largely regulated by stomatal behavior under the experimental conditions. Similarly, *E* was also positively associated with *g_s_*, reflecting the strong dependence of water loss on stomatal aperture.

In contrast, non-photochemical quenching (*NPQ*) exhibited strong negative correlations with photochemical parameters such as *qP* and *ΦPSII*, suggesting that when photochemical efficiency decreased, energy dissipation mechanisms were activated to protect the photosynthetic apparatus from excess excitation energy. This pattern is consistent with the activation of photoprotective responses commonly observed in grapevine under drought stress.

RWC showed relatively weak correlations with most gas exchange parameters, suggesting that the imposed water deficit primarily affected stomatal regulation rather than causing severe tissue dehydration. This finding supports the interpretation that plants experienced moderate physiological stress rather than critical water limitation.

Strong correlations were also observed among photosynthetic pigments (chlorophyll *a*, chlorophyll *b*, and total chlorophyll), reflecting the structural stability of the photosynthetic apparatus during the experimental period. Additionally, soluble proteins were positively associated with phenolic compounds (r = 0.74), indicating coordinated metabolic adjustments related to stress protection and antioxidant defense.

The multivariate correlation analysis highlights the coordinated regulation of gas exchange, photoprotection, and biochemical defense mechanisms involved in grapevine responses to water deficit and recovery.

## Conclusions and perspectives

This study demonstrated, in an integrated manner, that the autochthonous grapevine cultivars Touriga Franca (TF) and Tinto Cão (TC) exhibit distinct physiological strategies to cope with water deficit, revealing relevant adaptive responses under climate change scenarios. TF showed high functional plasticity, maintaining photosynthetic activity during drought, efficiently recovering after rehydration, and preserving stable pigment and antioxidant levels. In contrast, TC adopted a more conservative strategy, characterised by pronounced stomatal closure, increased accumulation of soluble proteins and phenolic compounds under stress, and enhanced leaf water retention. Although TC exhibited lower photosynthetic efficiency during water deficit, it activated strong defence mechanisms, conferring resilience under short-term drought events.

Comparative analysis indicates that TF has considerable potential for viticultural systems subject to prolonged water deficit, while TC may be more suitable for environments with lower water deficit, provided adequate agronomic management practices are applied. Beyond the direct implications for viticulture in the Douro Demarcated Region, these findings offer valuable insights for varietal selection and adaptation strategies, promoting sustainable production and water resource conservation. These findings reinforce the relevance of cultivar-specific physiological traits for guiding irrigation strategies and cultivar selection in Mediterranean viticultural regions facing increasing drought frequency and intensity.

For future research, it is recommended to extend the duration of water deficit treatments, conduct field trials in real conditions, and include additional regional cultivars, complemented by “omics” approaches (transcriptomics and metabolomics), to deepen the understanding of adaptive mechanisms to water stress. Such studies will support the development of integrated viticultural models that are technically sound, ecologically sustainable, and adapted to emerging climatic conditions.

## Data Availability

The raw data supporting the conclusions of this article will be made available by the authors, without undue reservation.
